# Glycaemic control trends in people with type 1 diabetes in Scotland 2004–2016

**DOI:** 10.1007/s00125-019-4900-7

**Published:** 2019-05-18

**Authors:** Colette Mair, Wahyu Wulaningsih, Anita Jeyam, Stuart McGurnaghan, Luke Blackbourn, Brian Kennon, Graham Leese, Robert Lindsay, Rory J. McCrimmon, John McKnight, John R. Petrie, Naveed Sattar, Sarah H. Wild, Nicholas Conway, Ian Craigie, Kenneth Robertson, Louise Bath, Paul M. McKeigue, Helen M. Colhoun, on behalf of the Scottish Diabetes Research Network (SDRN) Epidemiology Group

**Affiliations:** 1 MRC Institute of Genetic and Molecular Medicine, The University of Edinburgh, Western General Hospital, Crewe Road, Edinburgh, EH4 2XU UK; 2 Department of Diabetes, NHS Greater Glasgow & Clyde, Glasgow, UK; 3 Department of Public Health, NHS Fife, Kirkcaldy, UK; 4 Institute of Cardiovascular and Medical Sciences, University of Glasgow, Glasgow, UK; 5 Division of Molecular and Clinical Medicine, University of Dundee, Dundee, UK; 6 Metabolic Unit, Western General Hospital, Edinburgh, UK; 7 Usher Institute of Population Health Sciences and Informatics, University of Edinburgh, Edinburgh, UK; 8 NHS Tayside, Fife, Scotland UK; 9 GGC Children’s Diabetes Service, Glasgow, UK; 10 Royal Hospital for Children, Glasgow, UK; 11 NHS Lothian, Royal Hospital for Sick Children, Edinburgh, UK

**Keywords:** Additive mixed regression, Glycaemic control, HbA_1c_, Insulin, Type 1 diabetes

## Abstract

**Aims/hypothesis:**

The aim of this work was to examine whether glycaemic control has improved in those with type 1 diabetes in Scotland between 2004 and 2016, and whether any trends differed by sociodemographic factors.

**Methods:**

We analysed records from 30,717 people with type 1 diabetes, registered anytime between 2004 and 2016 in the national diabetes database, which contained repeated measures of HbA_1c_. An additive mixed regression model was used to estimate calendar time and other effects on HbA_1c_.

**Results:**

Overall, median (IQR) HbA_1c_ decreased from 72 (21) mmol/mol [8.7 (4.1)%] in 2004 to 68 (21) mmol/mol (8.4 [4.1]%) in 2016. However, all of the improvement across the period occurred in the latter 4 years: the regression model showed that the only period of significant change in HbA_1c_ was 2012–2016 where there was a fall of 3 (95% CI 1.82, 3.43) mmol/mol. The largest reductions in HbA_1c_ in this period were seen in children, from 69 (16) mmol/mol (8.5 [3.6]%) to 63 (14) mmol/mol (7.9 [3.4]%), and adolescents, from 75 (25) mmol/mol (9.0 [4.4]%) to 70 (23) mmol/mol (8.6 [4.3]%). Socioeconomic status (according to Scottish Index of Multiple Deprivation) affected the HbA_1c_ values: from the regression model, the 20% of people living in the most-deprived areas had HbA_1c_ levels on average 8.0 (95% CI 7.4, 8.9) mmol/mol higher than those of the 20% of people living in the least-deprived areas. However this difference did not change significantly over time. From the regression model HbA_1c_ was on average 1.7 (95% CI 1.6, 1.8) mmol/mol higher in women than in men. This sex difference did not narrow over time.

**Conclusions/interpretation:**

In this high-income country, we identified a modest but important improvement in HbA_1c_ since 2012 that was most marked in children and adolescents. These changes coincided with national initiatives to reduce HbA_1c_ including an expansion of pump therapy. However, in most people, overall glycaemic control remains far from target levels and further improvement is badly needed, particularly in those from more-deprived areas.

**Electronic supplementary material:**

The online version of this article (10.1007/s00125-019-4900-7) contains peer-reviewed but unedited supplementary material, which is available to authorised users.

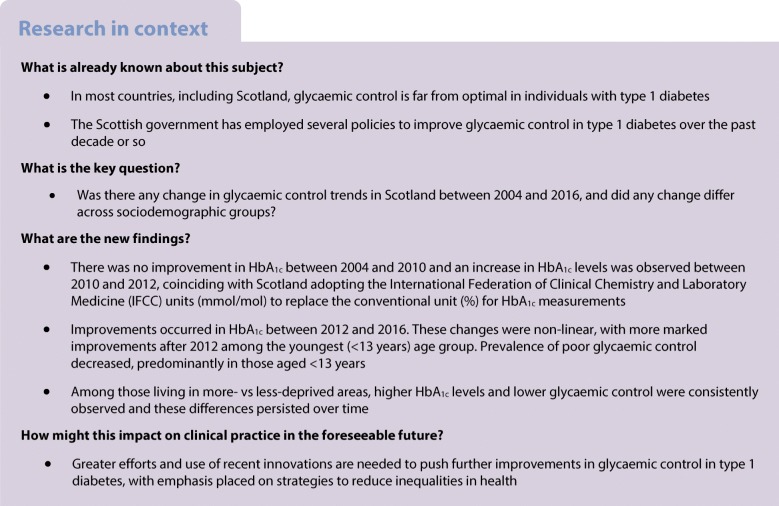



## Introduction

Type 1 diabetes is associated with a substantial reduction in life span [1] and a threefold increase in the rate of cardiovascular disease compared with individuals without diabetes and remains a common cause of end-stage renal disease and loss of vision [2]. Poor glycaemic control as indicated by HbA_1c_ is a key determinant of such complications and lowering HbA_1c_ reduces complications and prolongs survival rate [3]. However, achieving good levels of control remains a challenge in all countries. In an international study of type 1 diabetes in 19 countries in 2014, most people with type 1 diabetes had higher than recommended levels of HbA_1c_ [4]. Of those aged 15 years and more, median levels of HbA_1c_ were highest in Scotland.

As has happened to varying extents in other high-income countries, the publicly funded National Health Service (NHS) Scotland has employed several important changes to improve glycaemic control in type 1 diabetes in recent years [5]. Provision of insulin pumps has increased from 8.4% to 34.4% in those under 18 years and from 2.5% to 8.3% in adults between 2011 and 2016. In addition, during this period, policies were instituted to enhance access to early structured education and provision of psychological interventions and there was a slight expansion of continuous glucose monitoring (CGM). A national survey showed that the proportion of individuals with type 1 diabetes who achieved HbA_1c_ ≤58 mmol/mol (7.5%) in Scotland slightly improved from 21.5% in 2013 to 24.5% in 2016 [6]. This survey reports the overall population HbA_1c_ annually but does not test whether year-on-year changes represent significant trends or random fluctuations and does not explore detailed trends by age, sex or socioeconomic strata. Therefore, we analysed a nationwide diabetes register in Scotland enriched for patient characteristics and repeated measurements of HbA_1c_ to assess whether the significance of trends was beyond random fluctuations and to measure their consistency across age group, sex and socioeconomic strata. We sought evidence of whether healthcare innovations have had any impact on HbA_1c_ in this high-income country.

## Methods

### Study population

The Scottish Care Information-Diabetes Collaboration (SCI-Diabetes) database has been described [2]. This nationwide electronic healthcare record database captures registration of all patients assigned a diagnosis of diabetes in primary or secondary care healthcare information systems. Since 2004, the database has almost complete national coverage of all prevalent and incident cases of diabetes. All but five of 1076 general practices nationwide contributed data continuously over this period, yielding over 99.5% coverage of all diagnosed cases of diabetes. Type 1 diabetes was identified using information on age, drug prescription and clinical description of the type of diabetes. This approach has previously been validated in SCI-Diabetes against inpatient records, with greater than 99% accuracy. Those whose type of diabetes was not known were excluded. The study was approved by the Scotland A Research Ethics Committee, Privacy (Caldicott) Guardians for the 14 Scottish Health Boards and the Information Services Division (ISD) of NHS National Services Scotland Privacy Advisory Committee.

From SCI-Diabetes, we selected all patients alive with type 1 diabetes at any time from 2004 to 2016 with recorded age at diabetes diagnosis, sex, ethnic group, health board, the Scottish Index of Multiple Deprivation (SIMD) and date of birth and who had more than one measurement of HbA_1c_ (*N* = 30,717). Thus, in any 1 year the data comprise that from prevalent cases of type 1 diabetes alive and any newly incident cases arising in that year. The SIMD is a residential area-based proxy measure of individual socioeconomic status [7].

### Measurements of HbA_1c_ and other variables

HbA_1c_ was measured using a variety of clinical methods, all of which were aligned to the assay used in the DCCT. In Scotland, HbA_1c_ was recorded in % in earlier years then converted into the International Federation of Clinical Chemistry and Laboratory Medicine (IFCC) units (mmol/mol) in 2010–2011. We used all available data in the clinical record on HbA_1c_ measures throughout the study as the outcome of interest.

Data were categorised into six age groups at baseline (in years): <13, 13–18, 19–24, 25–44, 45–64 and >64. For each subsequent year they contributed data to the analysis, an individual may have been categorised into different age groups in the regression analysis as they aged.

‘Poor’ glycaemic control was defined as HbA_1c_ >75 mmol/mol (9%) and ‘good’ glycaemic control was defined as HbA_1c_ ≤58 mmol/mol (7.5%) in individuals aged ≤18 years and ≤53 mmol/mol (7%) in individuals aged >18 years as defined by the ADA [8]. We also considered the National Institute for Health and Care Excellence (NICE) guidelines for good glycaemic control of ≤48 mmol/mol (6.5%) [9].

### Statistical analysis

Medians and interquartile ranges were presented across years and separated by sex, SIMD category and age group. We used a fully flexible modelling approach with unrestrictive assumptions to capture non-linear trends in HbA_1c_ over time. This offered the potential to uncover hidden significant trends in HbA_1c_ rather than taking a group-based trajectory approach [10]. For this reason, changes in log transformed HbA_1c_ between 2004 and 2016 were assessed by fitting an additive mixed regression model with patient identifier as a random effect and a first-order autoregressive correlation structure to account for temporal dependencies in these data. This approach allowed us to capture non-linear trends in HbA_1c_ over time through regularised, non-parametric smooth functions, therefore relaxing any assumptions surrounding the nature of all relationships. We used the mgcv package version 1.8-28 in R [11] (downloaded from https://www.stats.bris.ac.uk/R/).

The model included age at diagnosis of diabetes, current age group, sex, ethnic group and SIMD band. We included interaction terms, or smooth functions, for current age group, sex and SIMD band over time in order to identify trends in HbA_1c_ in each category of these factors. Smooth functions were estimated by cubic regression splines. To account for seasonality [12], a smooth function for month was estimated by a cyclic penalised cubic regression spline to allow continuity between December and January in the following year. Significant periods of change were identified in each time series by estimating the rate of change along a grid of time points between 2004 and 2016. Bonferroni correction was used to maintain an overall significance level of 0.05.

We selected a grid of points and estimated the derivative (i.e. the gradient) of each smooth function at these points. This was repeated, increasing the chosen grid of time points by a small amount *ϵ* = 1*e*^−6^. We then compared the change in derivative (equivalent to comparing the second derivative) between the closely selected points. A positive change indicates the function is increasing, thereby identifying periods of significant increase, whereas a negative change indicates the function is decreasing and no change indicated the function is stationary [13].

In the multivariate regression model to test significance of calendar time trends, we adjusted for age, sex, SIMD band, age at onset of diabetes, health board and season. We used non-parametric bootstraps with replacement of fitted values in order to infer significant differences in change between age groups, sexes and SIMD bands.

## Results

Between 2004 and 2016 we had a median of 21 measurements per individual (interquartile range 15) across the 13 year period. In any given year the median number of HbA_1c_ measurements per individual varied from 1 to 2 (Table 1). The median duration of diabetes was 14 years in 2004 and 19 years in 2016. Within each year, between 0.048% and 0.01% of people were in the first year of diagnosis (Table 1).

**Table 1:** The distribution of duration and HbA_1c_ levels among people with type 1 diabetes in Scotland by year between 2004 and 2016

Variable	2004	2005	2006	2007	2008	2009	2010	2011	2012	2013	2014	2015	2016
HbA_1c_													
Median (IQR), mmol/mol	72 (21)	72 (21)	70 (22)	70 (22)	70 (20)	70 (20)	70 (22)	70 (22)	71 (21)	70 (22)	68 (21)	68 (21)	68 (21)
Median (IQR), %	8.7 (4.1)	8.7 (4.1)	8.6 (4.2)	8.6 (4.2)	8.6 (4)	8.6 (4)	8.6 (4.2)	8.6 (4.2)	8.6 (4.1)	8.6 (4.2)	8.4 (4.1)	8.4 (4.1)	8.4 (4.1)
Percentage of individuals within HbA_1c_ category													
>48 mmol/mol (6.5%)	96	96	96	95	95	95	95	96	96	95	94	95	95
>53 mmol/mol (7%)	90	91	90	90	90	90	90	91	91	90	88	88	89
>58 mmol/mol (7.5%)	82	82	82	81	80	81	81	81	83	81	78	78	79
>64 mmol/mol (8%)	70	70	69	68	68	68	68	69	70	67	63	63	64
>75 mmol/mol (9%)	42	42	41	40	40	40	40	41	42	39	36	36	36
>86 mmol/mol (10%)	21	21	21	21	20	20	20	21	22	20	18	18	18
Median number of HbA_1c_ measurements per individual	1	1	2	2	2	2	2	2	1	1	2	2	1
*N*	19,449	20,608	21,177	21,419	22,092	22,345	22,849	22,985	22,892	23,452	24,220	23,984	19,521
Median (IQR) duration of diabetes, years	14 (17)	14 (17)	15 (17)	15 (18)	16 (18)	16 (18)	16 (19)	17 (19)	17 (19)	17 (20)	18 (20)	18 (20)	19 (21)
Proportion of people in first year of diagnosis	0.043	0.043	0.043	0.048	0.046	0.045	0.047	0.048	0.047	0.044	0.043	0.031	0.010^a^

^a^Since 2016 is a partial year of data, the proportion diagnosed in this year is lower

IQR, interquartile range

The distribution of the characteristics of the population at the midpoint of each year studied were very stable across the time period studied, with the male sex making up 55% of the population in 2004 and 54% in 2016 and the median age of diabetes onset being 20 years in men/boys and 18 years in women/girls consistently across the period.

The study was carried out in accordance with the ethical principles in the Declaration of Helsinki as revised in 2008.

### Calendar time trends in HbA_1c_ across the population of Scotland with type 1 diabetes from 2004 to 2016

**Fig. 1:**
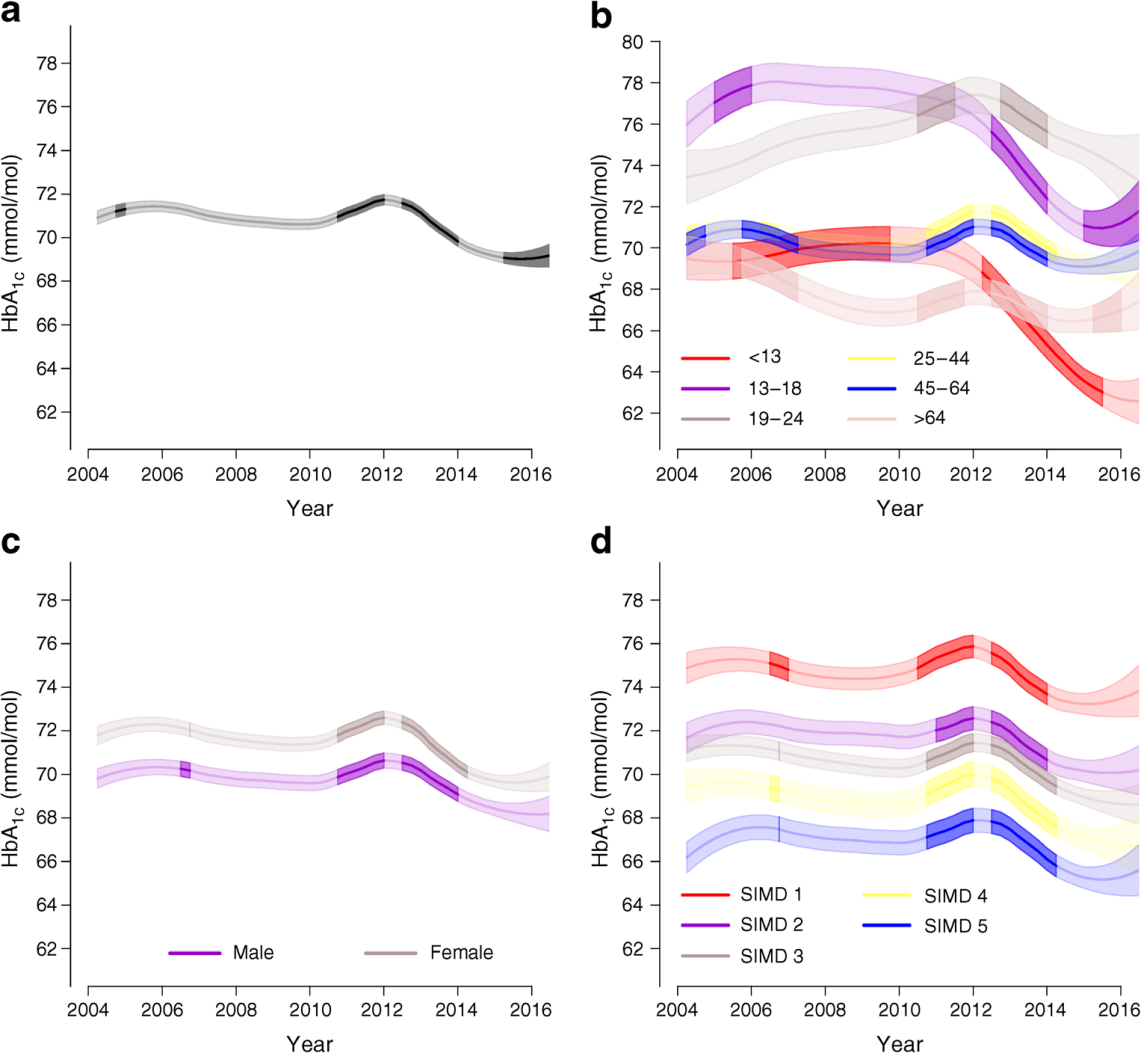
Estimated HbA_1c_ trajectories and 95% CI in all individuals (**a**) and stratified by age (**b**), sex (**c**) and SIMD band (where 1 is the most-deprived band) (**d**). Time periods in which significant changes occurred overall (**a**) and specifically for each stratum (**b**, **c**, **d**) are highlighted. Bonferroni correction was used to maintain an overall significance level of 0.05

**Fig. 2:**
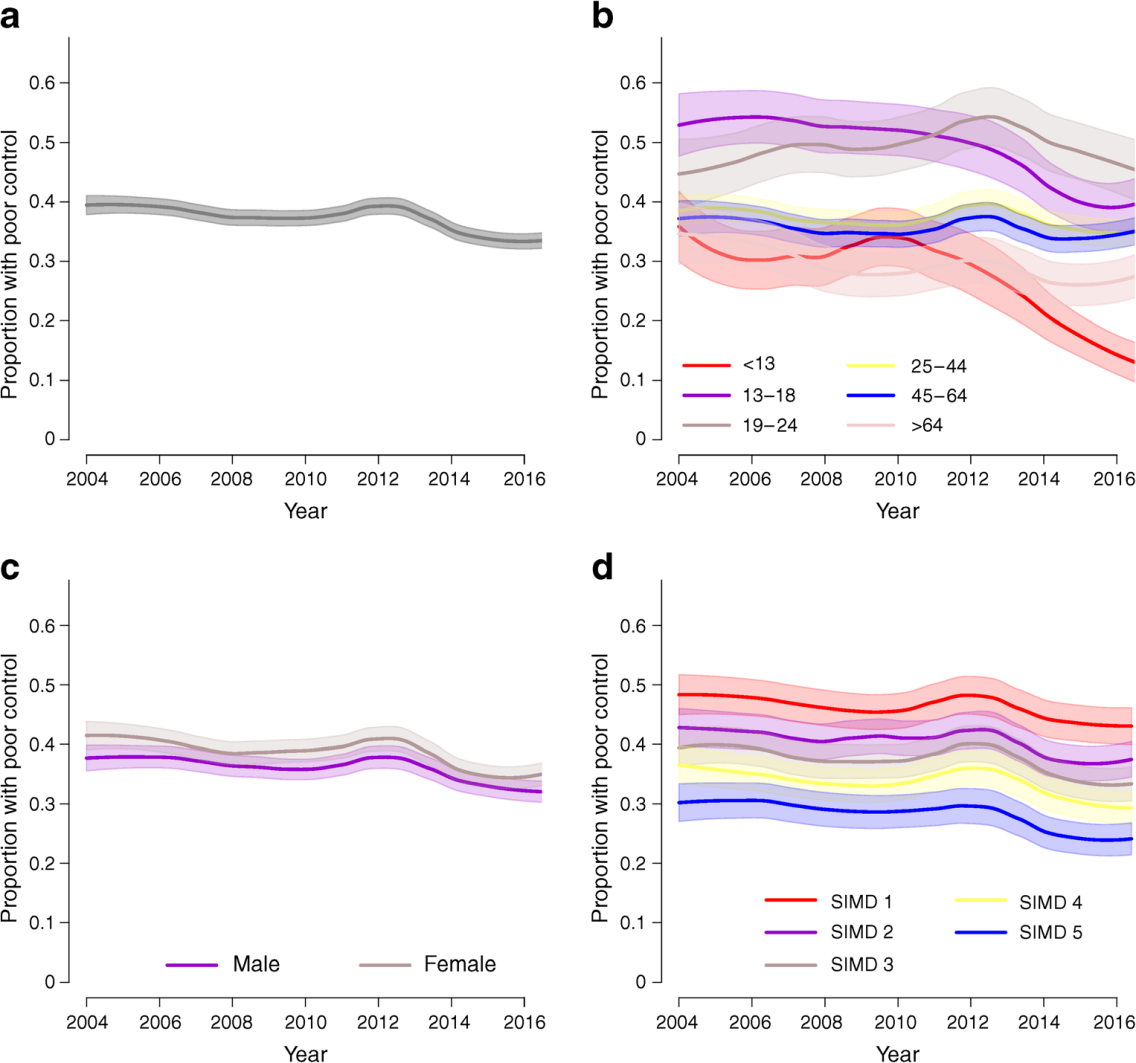
Estimated proportion and 95% CI of people with type 1 diabetes in Scotland with poor glycaemic control (**a**), and stratified by age (**b**), sex (**c**) and SIMD band (where 1 is the most-deprived band) (**d**). Poor control was defined as HbA_1c_ >75 mmol/mol (9%)

In the overall population, the median (IQR) HbA_1c_ fell from 72 (21) mmol/mol (8.7 [4.1]%) in 2004 to 68 (21) mmol/mol (8.4 [4.1]%) in 2016, a fall of six percentage points (Table 1, Fig. 1a). There was a substantial fall in the proportion of people with poor glycaemic control, defined as HbA_1c_ >75 mmol/mol (9%), from 42% in 2004 to 36% 2016 (Table 1, Fig. 2a). The proportion of people at target HbA_1c_ (≤53 mmol/mol [7%] in adults and ≤58 mmol/mol [7.5%] in children and adolescents) improved from 11% to 15% but only small proportions of people attained the NICE target of 48 mmol/mol (6.5%) at any time (4% in 2004 and 5% in 2016). There was a transient rise in HbA_1c_ during the period 2010–2012 (Fig. 1a). Note all figures use the modelled estimates and the confidence limits for the time trend from the models.

In a regression model combining data across the study period, variables associated with significant variation in HbA_1c_ included age, sex, age at diagnosis of type 1 diabetes, health board of treatment, SIMD band and season (electronic supplementary material [ESM] Table 1); HbA_1c_ was significantly lower in summer and higher in winter. Therefore, in the multivariate regression model, we adjusted for these variables. The calendar periods of significant change in HbA_1c_ at population level were 2010–2012 (Fig. 1a) where there was a rise of 1 (95% CI 0.8, 1.45) mmol/mol and then 2012–2016 where there was a fall of 3 (95% CI 1.82, 3.43) mmol/mol (Fig. 1a). Thus, all of the improvement across the period 2004–2016 occurred in the latter 4 years of the period.

### HbA_1c_ trends by age

From 2012, a significant decline in HbA_1c_ was seen in all age groups, with the most marked decline in the two younger age groups <13 years and 13–18 years (Fig. 1b and ESM Table 2). Median HbA_1c_ between 2012 and 2016 fell significantly from 69 (16) to 63 (14) mmol/mol (8.5 [3.6]% to 7.9 [3.4]%) and from 75 (25) to 70 (23) mmol/mol (9.0 [4.4]% to 8.6 [4.3]%) in these groups, respectively. Consistently, the proportion with poor glycaemic control (>75 mmol/mol [9%]) fell most in this time period in these two age groups, from 32% to 16% and from 51% to 42% respectively (ESM Table 2, Fig. 2b). However, across all age groups, the proportions with poor control remained high in 2016, being highest (48%) in those aged 19–24 years (Fig. 2b and ESM Table 2). The proportion of those aged <13 years and 13–18 years with good glycaemic control increased between 2012 and 2016 but in these, as with other age groups, still only a minority of people achieved target HbA_1c_ values, whether considering the NICE targets of 48 mmol/mol (6.5%) or age-specific targets of 53 and 58 mmol/mol (7% and 7.5%, respectively) (ESM Figs. 1b, 2b). Of note, the transient increase in HbA_1c_ in 2010–2012 was seen in all age groups 19 years and upwards (ESM Table 2).

### HbA_1c_ trends by sex

Women consistently had slightly higher average HbA_1c_ than men (ESM Table 3) with differences of 1–2 mmol/mol across the years. From the regression model the HbA_1c_ was on average 1.7 (95% CI 1.6, 1.8) mmol/mol higher in women than in men across the period. The increases in HbA_1c_ in 2010–2012 and the subsequent fall between 2012 and 2016 were of similar magnitude in both men and women, such that the sex difference persisted over time (Fig. 1c). Consistent with the median levels, women consistently had slightly but significantly higher proportions of poor glycaemic control and this sex difference persisted over time (Fig. 2c and ESM Table 3). There was little sex difference in the proportion of people who met targets across the time frame (ESM Figs. 1c, 2c, ESM Table 3).

### HbA_1c_ trends by area-level deprivation

Large socioeconomic differences in HbA_1c_ levels were observed. Those living in the most-deprived areas, indicated by the first band of SIMD (SIMD 1), had substantially higher HbA_1c_ levels across the period in comparison with the band living in the least-deprived areas (ESM Table 4). Using the regression model the 20% of people living in the most-deprived areas had HbA_1c_ levels on average 8.0 (95% CI 7.4, 8.9) mmol/mol higher than those of the 20% of people living in the least-deprived areas. These differences were apparent in all age groups (data not shown). The significant increases in HbA_1c_ in 2010–2012 and decreases in 2012–2015 were apparent within all SIMD bands (Fig. 1d), such that the difference in HbA_1c_ between SIMD bands did not change over time (ESM Table 4). Consistent with median levels of HbA_1c_, a much greater proportion of people living in SIMD 1 continued to have poor glycaemic control compared with people living in SIMD 5 (46% vs 26%) up to the end of the evaluation period in 2016 (Fig. 2d and ESM Table 4).

## Discussion

We present trends in glycaemic trajectories in type 1 diabetes for a national population across a 13 year period, showing a small overall improvement in HbA_1c_ levels between 2004 and 2016 of about 4 mmol/mol and a corresponding decrease in the percentage of those with poor glycaemic control. Nonetheless, by 2016, more than one-third of all those with type 1 diabetes still had poor glycaemic control and most did not achieve HbA_1c_ targets, particularly those in late adolescence/early adulthood. That noted, the largest improvement in control was seen in the two youngest age groups, which if such improvements are sustained over time is encouraging, given some evidence that those who develop diabetes younger also have the highest risks for adverse cardiovascular outcomes [14]. However, we also found large socioeconomic differentials in HbA_1c_ that did not alter in this time period.

We do not know which aspects of diabetes management may have altered HbA_1c_ during this period. However, the timing and larger reduction in younger people from 2012 is consistent with an impact of age-specific policy changes. A major policy change to quickly increase provision of insulin pumps in Scotland was introduced in 2011, and initially was mostly targeted towards children [6]. Although a recent study in England and Wales did not suggest any benefit in HbA_1c_ reduction with insulin pumps compared with multiple daily insulin injections in children and adolescents [15], other studies have reported improvements [16]. Apart from insulin pump policies, the larger improvement in children may have reflected other differences in services between paediatrics and adult clinics (e.g. the introduction of insulin pump therapy was accompanied by more widespread application of structured education). However, there have also been a number of focused initiatives among the 12 paediatric clinics that care for all of the paediatric population with type 1 diabetes in Scotland in this time period, including ensuring comprehensive education on carbohydrate counting and dynamic insulin dosing from diagnosis, regular meetings of the 12 leads from these centres at which key metrics on glycaemic control and policies are reviewed. Over the time period studied, there was no improvement in BMI or smoking, which show some relationship with HbA_1c_.

Although findings in older age groups were less marked, there were reductions in the prevalence of poor glycaemic control in all age groups. Apart from insulin pumps, the benefit of other measures to improve glycaemic control remain unclear. The Scottish Government introduced a 2 year funded study to support Psychology in Diabetes, Psychology and Diabetes (PiD-PaD) to improve self-management of diabetes [17] but this psychology support is still not widely available. Structured education, on the other hand, was recently shown to cost-effectively improve glycaemic control with or without insulin pump for adults [18]. Other potential contributing factors include the increasing availability of SCI-Diabetes data in 2011, allowing health centres to compare achievement of glycaemic control. In March 2014, the first national comparison of HbA_1c_ data for the 0–18 years age range appeared and the data have been discussed at the National Paediatric Diabetes Multidisciplinary Team annual meeting since then. Starting in 2014, there was a national campaign to standardise and tighten glycaemic targets for individuals with type 1 diabetes [17] and in January 2016, a national Scottish meeting set several key core targets, which have been cascaded across Scotland with the use of ‘Know your HbA_1c_ charts’. At present, we are unable to assess these specific measures across the datasets but future studies may look into types of insulin and change between regimens, as well as the emerging expansion of flash glucose monitoring (FGM). These trend data from this high-income country are encouraging, yet they also emphasise that even in such a resource-rich setting, wherein the NHS is free at the point of delivery and there is a concerted national policy, there remains an enormous challenge in achieving HbA_1c_ targets levels in most individuals with type 1 diabetes. It is worth noting the persistently poor glycaemic control in those aged 19–24 years. While transition from paediatric to adult care is rightly considered important, these data suggest a significant problem possibly initiated in but extending beyond the transition/transfer period. Strategies to improve control in this vulnerable age group must address issues of healthcare disengagement, including new models of care, greater accessibility and wider availability of services such as clinical psychology.

It would be interesting to evaluate whether other countries have achieved greater gains over this period. The Diabetes-Patienten-Verlaufsdokumentation (DPV) database in Germany and Austria showed that despite substantial improvements in pump availability and other care aspects expected to improve HbA_1c_, HbA_1c_ actually increased between 2002 and 2011 before falling thereafter [19]. Data from the Swedish National Diabetes Registry reveal that HbA_1c_ increased by 2 mmol/mol (2.3%) between 2007 and 2012 and decreased afterwards until 2017 [20]. Data from the USA show that mean HbA_1c_ levels were 66 mmol/mol (8.2%) in individuals enrolled into the T1D Exchange Clinic Network in 2010–2012, rising to 68 mmol/mol (8.4%) in the same individuals in 2013–2014 [21]. This increase was greatest among those aged 13–17 and 18–26 years. Also in the USA, the National Health and Nutrition Examination Survey showed an increasing proportion of individuals with HbA_1c_ ≤53 mmol/mol (7%) from 1999–2002 to 2003–2006 in adults with any diabetes, followed by a plateau until 2011–2014 [22]. Although mean HbA_1c_ values in individuals with type 1 diabetes in England seemed to be stable between 1998 and 2013 [23], the percentage of individuals achieving HbA_1c_ ≤58 mmol/mol (7.5%) decreased from 28.7% in 2009–2010 to 27.0% in 2011–2012 before increasing to 30% in 2016–2017 in England and Wales [24]. Increased cost sharing may have explained the plateau in glycaemic control attainment in the USA, whereas population changes may have contributed towards the temporary increases in HbA_1c_ in other countries. Changes in care process may also play a role as HbA_1c_ attainment varies across centres in Germany, Austria, England, Wales, USA, Sweden, Denmark and Norway [25].

We noted an increase in HbA_1c_ between 2010 and 2012 in most age groups, both sexes, all socioeconomic strata and all health boards. Of note, the denominator population in Scotland in our data was fairly stable during this time and such increase was seen across all age groups. A potential explanation for this increasing trend was the policy to adopt IFCC units (mmol/mol) to replace the conventional DCCT unit (%) for HbA_1c_ measurements. From June 2009, a dual reporting method with both the DCCT units and IFCC units was used in Scotland during a short adaptation period for both clinicians and patients before fully transitioning to IFCC units from October 2011 [26]. The impact of this change, particularly on patient care, remains unclear. Similar increases in mean HbA_1c_ that coincided with the IFCC standardisation have been reported in Sweden [20]. Therefore, our 2010–2012 findings may well have resulted from biases related to the method of HbA_1c_ reporting rather than real increases per se.

Despite the encouraging improvement in population HbA_1c_, our data showed that there are large persistent unchanging socioeconomic inequalities in HbA_1c_ across all age groups. In 2016, HbA_1c_ in the most-deprived residential category was around 8 mmol/mol (2.9%) higher compared with HbA_1c_ in the least-deprived category. To put this into context, the DCCT trial data suggest that a relative difference of 10% in HbA_1c_ may lead to a difference of 30–60% in microvascular complications of diabetes [27]. Although these data do not allow us to determine the cause of the differential we observed, we previously reported (in a subset of one-third of adults with type 1 diabetes in Scotland) that those living in more-deprived areas had a lower frequency of injections of insulin per day, lower pump use, lower numbers of glucose monitoring per day and were less likely to use carbohydrate counting [28] and by inference were less likely to have received structured education. Correspondingly, in other countries, HbA_1c_ has been reported to be higher among people of lower social class and lower educational attainment [29]. This may contribute to the socioeconomic inequalities of complications in type 1 diabetes, such as diabetic retinopathy and foot ulceration [30]. Our findings therefore prompt the need to ensure the achievement of adequate glycaemic control equally across the spectrum of socioeconomic status. It is particularly important to ensure that recent innovations expected to improve glucose management in diabetes in future, such as CGM and FGM, and widening coverage of pump availability, reach all of those in need across socioeconomic strata.

We also noted that sex differences in HbA_1c_ levels persisted over time, with better glycaemic control in men than in women. Higher HbA_1c_ in girls, compared with boys, at time of first diagnosis with type 1 diabetes have been reported [31]. The higher HbA_1c_ levels in women may underestimate the true difference, since anaemia, more common in women is expected to lower HbA_1c_ levels [32]. The magnitude of these sex differences is slight in comparison with the magnitude of the socioeconomic differences.

The strength of our study lies in the population-based data (99.5% coverage) with repeated measures of HbA_1c_ for over a decade, which allowed us to estimate long-term glycaemic trends. A limitation is our use of an area-based rather than individual measures of socioeconomic status. In addition, we do not yet have sufficient individual-level data on new insulin delivery systems and other innovations, including flash monitoring and structured education in diabetes management, to enable a direct assessment of the impact within person before and after changes in treatment. This will be the subject of future research when the information becomes available.

### Conclusion

Small but meaningful improvements were seen in glycaemic control among people with type 1 diabetes in Scotland between 2004 and 2016 with, notably, the improvements being greatest in children and adolescents, groups at highest excess risk of premature death. Large socioeconomic differentials in HbA_1c_ persisted across the period. The prevalence of poor glycaemic control remain high and guideline HbA_1c_ targets are elusive for most. Clearly greater action and use of recent innovations is needed to push further improvements in glycaemic control in type 1 diabetes. In particular, it will be important to monitor the impact of specific person-level interventions including flash monitors, widening pump use and potential use of additional oral glucose-lowering drugs, as well as innovations in other aspects of care, including service organisation innovations such as digital technologies.

## Electronic supplementary material

ESM (PDF 473 kb)

## Data Availability

We do not have governance permissions to share individual-level data on which these analyses were conducted. However, for any bona fide requests to audit the validity of the analyses, the verifiable research pipeline which we operate means that researchers can make a request to the corresponding author to view the analyses being run and the same tabulations resulting.

## Abbreviations

CGM: Continuous glucose monitoring
FGM: Flash glucose monitoring
NHS: National Health Service
IFCC: International Federation of Clinical Chemistry and Laboratory Medicine
NICE: National Institute for Health and Care Excellence
SCI-Diabetes: Scottish Care Information-Diabetes Collaboration
SIMD: Scottish Index of Multiple Deprivation

## References

[1] Livingstone SJ, Levin D, Looker HC (2015). Estimated life expectancy in a Scottish cohort with type 1 diabetes, 2008-2010. JAMA.

[2] Livingstone SJ, Looker HC, Hothersall EJ (2012). Risk of cardiovascular disease and total mortality in adults with type 1 diabetes: Scottish registry linkage study. PLoS Med.

[3] Orchard TJ, Nathan DM, Zinman B (2015). Association between 7 years of intensive treatment of type 1 diabetes and long-term mortality. JAMA.

[4] McKnight JA, Wild SH, Lamb MJE (2015). Glycaemic control of type 1 diabetes in clinical practice early in the 21st century: an international comparison. Diabet Med.

[5] Director-General Health & Social Care and Chief Executive NHS Scotland (2012) Insulin pump therapy for people with type 1 diabetes. https://www.sehd.scot.nhs.uk/mels/cel2012_04.pdf. Accessed November 2018

[6] Scottish Diabetes Survey Monitoring Group (2016) Scottish Diabetes Survey 2016. http://www.diabetesinscotland.org.uk/Publications/Scottish%20Diabetes%20Survey%202016.pdf. Accessed November 2018

[7] Govan L, Maietti E, Torsney B (2012). The effect of deprivation and HbA_1c_ on admission to hospital for diabetic ketoacidosis in type 1 diabetes. Diabetologia.

[8] Bakst H (2018). Introduction: Standards of Medical Care in Diabetes—2018. Diabetes Care.

[9] National Institute for Health and Care Excellence (2008) Continuous subcutaneous insulin infusion for the treatment of diabetes mellitus. https://www.nice.org.uk/guidance/ta151. Accessed November 2018

[10] Nagin DS, Jones BL, Passos VL, Tremblay RE (2018). Group-based multi-trajectory modeling. Stat Methods Med Res.

[11] Wood SN (2017). Generalized additive models: an introduction with R.

[12] Gikas A, Sotiropoulos A, Pastromas V, Papazafiropoulou A, Apostolou O, Pappas S (2009). Seasonal variation in fasting glucose and HbA1c in patients with type 2 diabetes. Prim Care Diabetes.

[13] Simpson G (2014) Identifying periods of change in time series with GAMs. https://www.fromthebottomoftheheap.net/2014/05/15/identifying-periods-of-change-with-gams/. Accessed November 2018

[14] Miller RG, Costacou T, Orchard TJ (2019). Risk factor modeling for cardiovascular disease in type 1 diabetes in the Pittsburgh Epidemiology of Diabetes Complications (EDC) Study: a comparison to the Diabetes Control and Complications Trial/Epidemiology of Diabetes Interventions and Complications Study. Diabetes.

[15] Blair J, McKay A, Ridyard C (2018). Continuous subcutaneous insulin infusion versus multiple daily injections in children and young people at diagnosis of type 1 diabetes: the SCIPI RCT. Health Technol Assess.

[16] Evans-Cheung TC, Campbell F, Yong J, Parslow RC, Feltbower RG (2019). HbA1c values and hospital admissions in children and adolescents receiving continuous subcutaneous insulin infusion therapy. Diabet Med.

[17] McKnight JA (2015). The Scottish Diabetes Improvement Plan 2014. Br J Diabetes.

[18] Pollard DJ, Brennan A, Dixon S (2018). Cost-effectiveness of insulin pumps compared with multiple daily injections both provided with structured education for adults with type 1 diabetes: a health economic analysis of the Relative Effectiveness of Pumps over Structured Education (REPOSE) randomised controlled trial. BMJ Open.

[19] Bohn B, Kerner W, Seufert J (2016). Trend of antihyperglycaemic therapy and glycaemic control in 184,864 adults with type 1 or 2 diabetes between 2002 and 2014: analysis of real-life data from the DPV registry from Germany and Austria. Diabetes Res Clin Pract.

[20] Nordin G (2018). Accuracy of HbA1c as monitored by external quality assessment and compared with patient mean values. J Diabetes Sci Technol.

[21] Miller KM, Foster NC, Beck RW (2015). Current state of type 1 diabetes treatment in the U.S.: updated data from the T1D Exchange Clinic Registry. Diabetes Care.

[22] Carls G, Huynh J, Tuttle E, Yee J, Edelman SV (2017). Achievement of glycated hemoglobin goals in the US remains unchanged through 2014. Diabetes Ther.

[23] Zhong VW, Juhaeri J, Cole SR (2017). Incidence and trends in hypoglycemia hospitalization in adults with type 1 and type 2 diabetes in England, 1998–2013: a retrospective cohort study. Diabetes Care.

[24] NHS Digital (2018) National Diabetes Audit. https://digital.nhs.uk/data-and-information/clinical-audits-and-registries/national-diabetes-audit. Accessed November 2018

[25] Charalampopoulos D, Hermann JM, Svensson J (2018). Exploring variation in glycemic control across and within eight high-income countries: a cross-sectional analysis of 64,666 children and adolescents with type 1 diabetes. Diabetes Care.

[26] NHS Scotland (2009) HbA_1c_ standardisation for laboratory professionals. http://www.diabetesinscotland.org.uk/Publications/HbA1c_Lab_leaflet_0509.pdf. Accessed November 2018

[27] The Diabetes Control and Complications Trial Research Group (1995). The relationship of glycemic exposure (HbA_1c_) to the risk of development and progression of retinopathy in the Diabetes Control and Complications Trial. Diabetes.

[28] Akbar T, McGurnaghan S, Palmer CNA et al (2016) Cohort profile: Scottish Diabetes Research Network Type 1 Bioresource Study (SDRNT1BIO). Int J Epidemiol 46(3):796–769i. 10.1093/ije/dyw152 10.1093/ije/dyw152 PMC5582633 28338705

[29] Lindner LME, Rathmann W, Rosenbauer J (2018). Inequalities in glycaemic control, hypoglycaemia and diabetic ketoacidosis according to socio-economic status and area-level deprivation in type 1 diabetes mellitus: a systematic review. Diabet Med.

[30] Low L, Law JP, Hodson J, McAlpine R, O’Colmain U, MacEwen C (2015). Impact of socioeconomic deprivation on the development of diabetic retinopathy: a population-based, cross-sectional and longitudinal study over 12 years. BMJ Open.

[31] Hanberger L, Åkesson K, Samuelsson U (2014). Glycated haemoglobin variations in paediatric type 1 diabetes: the impact of season, gender and age. Acta Paediatr.

[32] Sinha N, Mishra TK, Singh T, Gupta N (2012). Effect of iron deficiency anemia on hemoglobin A1c levels. Ann Lab Med.

