# Supplementary material for: Glycaemic control trends in people with type 1 diabetes in Scotland 2004–2016
Source: Diabetologia. 2019 May 18;62(8):1375–84. doi: 10.1007/s00125-019-4900-7 (PMC6647722; doi:10.1007/s00125-019-4900-7)

ESM Table 1: Estimated fixed effect regression coefficient estimates for factors associated with HbA_{1c}.

	Estimate	Std. Error	t value	Pr(>|t|)
**(Intercept)**	67.7516	0.0047	899.2741	<0.0001
**Age at Onset**	10.9996	0.0001	-4.4728	<0.0001
**Gender female**	1.0165	0.0021	7.6055	<0.0001
**Health board authority 1**	1.0278	0.0082	3.3396	0.0008
**Health board authority 2**	1.0122	0.0057	2.1413	0.0322
**Health board authority 3**	1.0146	0.0056	2.5942	0.0095
**Health board authority 4**	1.0104	0.0048	2.1642	0.0305
**Health board authority 5**	1.0188	0.0063	2.9495	0.0032
**Health board authority 6**	1.0213	0.0049	4.3136	<0.0001
**Health board authority 7**	1.0691	0.0051	13.0195	<0.0001
**Health board authority 8**	0.9784	0.0174	-1.2559	0.2091
**Health board authority 9**	0.9947	0.0048	-1.1022	0.2704
**Health board authority 10**	1.0295	0.0057	5.1017	<0.0001
**Health board authority 11**	1.0434	0.0059	7.2128	<0.0001
**Health board authority 12**	1.0119	0.0142	0.8383	0.4019
**Health board authority 13**	1.0132	0.0072	1.8365	0.0663
**Health board authority 14**	0.9691	0.0164	-1.9131	0.0557
**SIMD 2**	0.9665	0.0034	-10.0977	<0.0001
**SIMD 3**	0.9813	0.0033	-5.6549	<0.0001
**SIMD 4**	0.9797	0.0034	-6.0889	<0.0001
**SIMD 5**	0.9662	0.0035	-9.7589	<0.0001
**Age band 13-18**	1.0595	0.0019	31.1203	<0.0001
**Age band 19-24**	1.0564	0.0024	22.7262	<0.0001
**Age band 25-44**	1.0139	0.0027	5.1816	<0.0001
**Age band 45-64**	1.0146	0.0029	5.0369	<0.0001
**Age band ≥65**	0.9913	0.0035	-2.5177	0.0118

Note: Health board authority was separated in fourteen dummy variables. In this table, Gender female shows females in comparison to males. Age band 13-18, Age band 19-24, Age band 25-44, Age band 45-64, Age band >64 are dummy variables with children (age band <13) set as the baseline category. SIMD 2, SIMD 3, SIMD 4 and SIMD 5 are dummy variables with SIMD 1 set as the baseline category

ESM Table 2: The distribution of HbA_{1c} levels among people with type 1 diabetes in Scotland by year between 2004 and 2016 by age group.

**Age Group**		**2004**	**2005**	**2006**	**2007**	**2008**	**2009**	**2010**	**2011**	**2012**	**2013**	**2014**	**2015**	**2016**
Age <13	Median (IQR) Mmol/mol	69 (17)	68 (16)	69 (16)	69 (15)	69 (15)	70 (16)	69 (16)	69 (16)	69 (16)	66 (17)	64 (16)	63 (15)	63 (14)
	Median (IQR) %	8.5 (3.7)	8.4 (3.6)	8.5 (3.6)	8.5 (3.5)	8.5 (3.5)	8.6 (3.6)	8.5 (3.6)	8.5 (3.6)	8.5 (3.6)	8.2 (3.7)	8 (3.6)	7.9 (3.5)	7.9 (3.4)
	>48mmol/mol (%) (6.5%)	97	97	98	98	98	97	98	98	97	95	95	94	97
	>53mmol/mol (%) (7%)	92	92	94	94	95	94	93	93	93	89	86	86	89
	>58mmol/mol (%) (7.5%)	84	82	84	84	87	86	85	85	83	78	73	71	74
	>64mmol/mol (%) (8%)	69	68	70	69	72	73	71	69	67	60	53	49	50
	>75mmol/mol (%) (9%)	37	31	35	33	35	39	35	34	32	26	21	17	16
	>86mmol/mol (%) (10%)	15	12	14	14	12	14	12	12	11	8	7	6	4
	N	1180	1188	1203	1184	1185	1205	1205	1208	1117	1258	1426	1355	1120
Age 13-18	Median (IQR)	77 (24)	77 (26)	77 (26)	77 (26)	76 (25.5)	77 (26)	76 (26)	76 (26)	75 (25)	74 (25)	71 (25)	70 (25)	70 (23)
	Median (IQR) %	9.2 (4.3)	9.2 (4.5)	9.2 (4.5)	9.2 (4.5)	9.1 (4.4)	9.2 (4.5)	9.1 (4.5)	9.1 (4.5)	9 (4.4)	8.9 (4.4)	8.6 (4.4)	8.6 (4.4)	8.6 (4.3)
	>48mmol/mol (%) (6.5%)	97	98	98	98	97	98	97	98	97	96	95	95	96
	>53mmol/mol (%) (7%)	94	95	95	94	95	94	94	94	94	92	90	90	92
	>58mmol/mol (%) (7.5%)	89	89	90	89	88	89	88	88	87	85	81	81	83
	>64mmol/mol (%) (8%)	81	80	80	80	79	79	79	78	77	73	68	67	68
	>75mmol/mol (%) (9%)	56	56	56	56	55	55	54	53	51	48	43	41	42
	>86mmol/mol (%) (10%)	33	34	35	36	33	34	33	33	30	29	25	24	23
	N	1710	1795	1805	1812	1817	1817	1897	1888	1800	1945	2081	1982	1605
Age 19-24	Median (IQR)	73.5 (25)	74 (24)	75 (27)	75 (26)	75 (27)	75 (27)	76 (28)	76 (29)	79 (30)	76 (28)	75 (30)	75 (28)	73 (27)

Age Group		2004	2005	2006	2007	2008	2009	2010	2011	2012	2013	2014	2015	2016
	Median (IQR) %	8.9 (4.4)	8.9 (4.3)	9 (4.6)	9 (4.5)	9 (4.6)	9 (4.6)	9.1 (4.7)	9.1 (4.8)	9.4 (4.9)	9.1 (4.7)	9 (4.9)	9 (4.7)	8.8 (4.6)
	>48mmol/mol (%) (6.5%)	94	95	96	96	96	96	96	97	97	96	96	96	95
	>53mmol/mol (%) (7%)	89	91	91	92	93	92	92	92	93	92	91	91	91
	>58mmol/mol (%) (7.5%)	82	84	84	85	86	85	85	86	88	86	84	84	84
	>64mmol/mol (%) (8%)	72	74	74	75	76	75	76	77	79	77	73	73	71
	>75mmol/mol (%) (9%)	47	49	51	52	51	51	53	54	58	54	51	50	48
	>86mmol/mol (%) (10%)	27	28	31	32	31	33	34	35	38	34	33	32	29
	N	1699	1859	1895	1963	2056	2057	2183	2177	2167	2131	2220	2138	1550
Age 25-44	Median (IQR)	70 (22)	70 (22)	70 (23)	69 (23)	69 (21)	69 (21)	69 (21.2)	70 (23)	71 (23)	69 (23)	68 (24)	68 (24)	68 (23)
	Median (IQR) %	8.6 (4.2)	8.6 (4.2)	8.6 (4.3)	8.5 (4.3)	8.5 (4.1)	8.5 (4.1)	8.5 (4.1)	8.6 (4.3)	8.6 (4.3)	8.5 (4.3)	8.4 (4.3)	8.4 (4.3)	8.4 (4.3)
	>48mmol/mol (%) (6.5%)	95	96	95	94	94	94	94	95	95	95	93	93	93
	>53mmol/mol (%) (7%)	90	90	89	88	88	88	88	89	90	89	86	86	86
	>58mmol/mol (%) (7.5%)	80	81	80	78	78	78	78	79	81	79	75	76	76
	>64mmol/mol (%) (8%)	68	69	67	66	65	65	65	67	69	65	62	62	62
	>75mmol/mol (%) (9%)	41	42	40	39	39	38	39	40	43	40	37	37	37
	>86mmol/mol (%) (10%)	20	21	20	20	20	20	20	22	23	22	20	20	20
	N	7831	8121	8183	8070	8258	8174	8236	8048	7870	7806	7946	7786	5997
Age 45-64	Median (IQR)	70 (19)	70 (19)	70 (19)	69 (19)	69 (19)	69 (19)	69 (19)	70 (20)	71 (20)	69 (19)	69 (20)	69 (20)	69 (19)
	>48mmol/mol (%) (6.5%)	96	96	96	95	95	96	96	96	97	96	95	95	95
	>53mmol/mol (%) (7%)	91	91	91	90	90	90	91	91	92	91	89	90	90
	>58mmol/mol (%) (7.5%)	82	82	81	81	80	81	81	82	84	82	80	81	81

Age Group		2004	2005	2006	2007	2008	2009	2010	2011	2012	2013	2014	2015	2016
	>64mmol/mol (%) (8%)	70	70	68	68	67	67	67	69	71	67	65	67	66
	>75mmol/mol (%) (9%)	40	40	39	38	37	37	37	39	40	38	36	38	37
	>86mmol/mol (%) (10%)	18	19	18	17	16	17	17	19	19	18	17	18	17
	N	5684	6171	6535	6784	7167	7428	7671	7888	8022	8216	8485	8521	7006
Age >64	Median (IQR)	69 (19)	69 (18)	68 (21)	67 (20)	66 (20)	66 (20)	67 (20)	68 (20)	68 (18)	67 (19)	66 (19)	66 (18)	67 (19)
	Median (IQR) %	8.5 (3.9)	8.5 (3.8)	8.4 (4.1)	8.3 (4)	8.2 (4)	8.2 (4)	8.3 (4)	8.4 (4)	8.4 (3.8)	8.3 (3.9)	8.2 (3.9)	8.2 (3.8)	8.3 (3.9)
	>48mmol/mol (%) (6.5%)	95	95	95	94	94	93	94	95	95	95	94	94	95
	>53mmol/mol (%) (7%)	89	89	89	87	86	86	86	88	89	88	86	87	88
	>58mmol/mol (%) (7.5%)	79	79	78	76	74	74	74	76	80	76	74	75	76
	>64mmol/mol (%) (8%)	66	66	63	62	59	59	60	62	63	61	57	59	61
	>75mmol/mol (%) (9%)	37	35	35	33	30	30	30	33	33	31	28	29	30
	>86mmol/mol (%) (10%)	16	14	14	13	13	12	13	13	13	12	11	12	12
	N	1836	2049	2160	2197	2225	2272	2315	2404	2530	2669	2787	2837	2393

People are included for all the age bands they inhabited within any calendar year. Since a small number from one age band to another within a given calendar year, they contribute to both age bands and are thus counted in the N for each of those age bands.

ESM Table 3: The distribution of HbA_{1c} levels between 2004 and 2016 by sex

Gender		2004	2005	2006	2007	2008	2009	2010	2011	2012	2013	2014	2015	2016
Male	Median (IQR) mmol/mol	70 (20)	70 (20)	70 (20)	69 (20)	69 (21)	69 (20)	69 (20)	70 (20)	70 (22)	69 (21)	68 (21)	68 (21)	68 (20)
	Median (IQR) %	8.6 (4)	8.6 (4)	8.6 (4)	8.5 (4)	8.5 (4.1)	8.5 (4)	8.5 (4)	8.6 (4)	8.6 (4.2)	8.5 (4.1)	8.4 (4.1)	8.4 (4.1)	8.4 (4)
	>48mmol/mol (%) (6.5%)	96	96	96	95	95	95	95	96	96	95	95	95	95
	>53mmol/mol (%) (7%)	90	91	90	89	89	89	90	90	91	90	88	89	89
	>58mmol/mol (%) (7.5%)	81	82	81	80	79	80	80	81	83	80	78	79	79
	>64mmol/mol (%) (8%)	69	69	68	67	67	67	67	68	69	66	63	64	63
	>75mmol/mol (%) (9%)	40	40	40	39	39	38	38	40	41	38	35	35	35
	>86mmol/mol (%) (10%)	19	20	20	19	19	19	19	20	21	19	18	17	17
	N	10644	11354	11699	11821	12223	12345	12628	12658	12619	12896	13336	13122	10511
Female	Median (IQR) mmol/mol	72 (22)	72 (22)	72 (21)	70 (23)	70 (22)	70 (22)	70 (22)	72 (22)	72 (22)	70 (22)	68 (23)	69 (22)	69 (22)
	Median (IQR) %	8.7 (4.2)	8.7 (4.2)	8.7 (4.1)	8.6 (4.3)	8.6 (4.2)	8.6 (4.2)	8.6 (4.2)	8.7 (4.2)	8.7 (4.2)	8.6 (4.2)	8.4 (4.3)	8.5 (4.2)	8.5 (4.2)
	>48mmol/mol (%) (6.5%)	96	96	96	96	96	96	96	96	96	95	94	94	95
	>53mmol/mol (%) (7%)	90	91	91	90	90	90	90	91	91	90	87	88	88
	>58mmol/mol (%) (7.5%)	82	83	82	82	81	81	81	82	84	81	77	78	79
	>64mmol/mol (%) (8%)	71	71	70	69	69	69	69	70	71	68	63	63	64
	>75mmol/mol (%) (9%)	44	44	43	42	41	41	42	43	44	40	37	37	37
	>86mmol/mol (%) (10%)	22	22	22	22	21	21	21	23	23	21	19	20	19
	N	8805	9254	9478	9598	9869	10000	10221	10327	10273	10556	10884	10862	9010

ESM Table 4: The distribution of HbA_{1c} levels between 2004 and 2016 by SIMD quintile

SIMD		2004	2005	2006	2007	2008	2009	2010	2011	2012	2013	2014	2015	2016
SIMD 1	Median (IQR) mmol/mol	75 (24)	75 (24)	75 (23)	74 (24)	74 (23)	74 (24)	74 (24)	74 (24)	75 (24)	74 (25)	73 (25)	73 (24)	73 (24)
	Median (IQR) %	9 (4.3)	9 (4.3)	9 (4.3)	8.9 (4.3)	8.9 (4.3)	8.9 (4.3)	8.9 (4.3)	8.9 (4.3)	9 (4.3)	8.9 (4.4)	8.8 (4.4)	8.8 (4.3)	8.8 (4.3)
	>48mmol/mol (%) (6.5%)	96	97	96	96	96	96	96	97	97	96	95	96	96
	>53mmol/mol (%) (7%)	92	93	92	91	91	91	92	93	93	92	90	91	91
	>58mmol/mol (%) (7.5%)	85	86	85	84	84	83	84	86	87	84	82	83	83
	>64mmol/mol (%) (8%)	75	76	75	75	74	73	73	75	76	73	70	72	72
	>75mmol/mol (%) (9%)	51	50	51	49	49	48	49	50	51	48	46	46	46
	>86mmol/mol (%) (10%)	28	29	28	28	27	26	27	29	30	28	25	26	26
	N	3806	4030	4162	4225	4355	4357	4483	4520	4485	4517	4705	4653	3687
SIMD 2	Median (IQR) mmol/mol	73 (21)	72 (22)	72 (22)	72 (22)	72 (22)	72 (23)	72 (22)	72 (23)	73 (22)	71 (22)	70 (22)	70 (23)	70 (21)
	Median (IQR) %	8.8 (4.1)	8.7 (4.2)	8.7 (4.2)	8.7 (4.2)	8.7 (4.2)	8.7 (4.3)	8.7 (4.2)	8.7 (4.3)	8.8 (4.2)	8.6 (4.2)	8.6 (4.2)	8.6 (4.3)	8.6 (4.1)
	>48mmol/mol (%) (6.5%)	96	97	96	96	96	95	95	96	96	95	95	95	95
	>53mmol/mol (%) (7%)	90	92	91	91	91	91	90	91	92	90	89	89	90
	>58mmol/mol (%) (7.5%)	83	84	83	82	82	83	82	82	85	82	79	80	82
	>64mmol/mol (%) (8%)	73	72	71	71	71	72	70	71	73	69	67	67	68
	>75mmol/mol (%) (9%)	45	44	45	44	43	44	43	44	45	42	39	40	39
	>86mmol/mol (%) (10%)	23	22	23	23	22	24	23	23	24	22	20	21	20
	N	4040	4218	4354	4372	4494	4549	4648	4669	4672	4766	4897	4859	3904
SIMD 3	Median (IQR) mmol/mol	72 (21)	72 (21)	70 (19)	70 (22)	70 (20)	69 (20)	70 (20)	70 (22)	72 (22)	70 (21)	69 (22)	68 (21)	69 (20)
	Median (IQR) %	8.7 (4.1)	8.7 (4.1)	8.6 (3.9)	8.6 (4.2)	8.6 (4)	8.5 (4)	8.6 (4)	8.6 (4.2)	8.7 (4.2)	8.6 (4.1)	8.5 (4.2)	8.4 (4.1)	8.5 (4)
	>48mmol/mol (%) (6.5%)	96	96	96	96	96	95	96	96	97	96	95	95	95
	>53mmol/mol (%) (7%)	91	92	91	91	90	90	91	91	92	90	89	89	90
	>58mmol/mol (%) (7.5%)	83	84	83	82	80	82	81	82	84	82	78	79	80
	>64mmol/mol (%) (8%)	71	72	70	68	67	68	69	69	71	67	63	63	64

SIMD		2004	2005	2006	2007	2008	2009	2010	2011	2012	2013	2014	2015	2016
	>75mmol/mol (%) (9%)	42	42	41	40	40	39	39	42	43	39	37	35	36
	>86mmol/mol (%) (10%)	20	20	20	20	20	20	19	21	22	20	19	18	18
	N	4078	4358	4467	4514	4662	4742	4831	4814	4774	4915	5063	5015	4100
SIMD 4	Median (IQR) mmol/mol	69 (19)	69 (19)	69 (19)	69 (20)	68 (19)	68 (19)	68.5 (20)	69 (19)	70 (20)	69 (20)	67 (20)	67 (20)	67 (20)
	Median (IQR) %	8.5 (3.9)	8.5 (3.9)	8.5 (3.9)	8.5 (4)	8.4 (3.9)	8.4 (3.9)	8.5 (4)	8.5 (3.9)	8.6 (4)	8.5 (4)	8.3 (4)	8.3 (4)	8.3 (4)
	>48mmol/mol (%) (6.5%)	96	96	96	95	96	96	95	96	96	96	95	94	94
	>53mmol/mol (%) (7%)	90	90	90	89	90	89	89	91	91	90	88	87	87
	>58mmol/mol (%) (7.5%)	81	81	80	79	79	79	79	80	82	80	77	76	76
	>64mmol/mol (%) (8%)	68	68	67	65	65	65	65	67	68	65	61	60	61
	>75mmol/mol (%) (9%)	39	38	37	37	36	35	36	38	39	36	33	33	32
	>86mmol/mol (%) (10%)	18	17	18	17	17	16	17	18	18	17	15	15	14
	N	3927	4156	4292	4305	4455	4542	4640	4665	4687	4792	4955	4946	4042
SIMD 5	Median (IQR) mmol/mol	68 (20)	68 (20)	67 (20)	67 (20)	67 (19)	67 (19)	67 (20)	67 (20)	67 (19)	66 (19)	65 (19)	65 (19)	65 (18)
	Median (IQR) %	8.4 (4)	8.4 (4)	8.3 (4)	8.3 (4)	8.3 (3.9)	8.3 (3.9)	8.3 (4)	8.3 (4)	8.3 (3.9)	8.2 (3.9)	8.1 (3.9)	8.1 (3.9)	8.1 (3.8)
	>48mmol/mol (%) (6.5%)	94	95	94	94	93	94	95	95	95	94	92	93	93
	>53mmol/mol (%) (7%)	88	88	88	87	86	87	87	88	89	87	84	85	85
	>58mmol/mol (%) (7.5%)	77	77	76	76	75	75	76	76	78	75	72	72	72
	>64mmol/mol (%) (8%)	63	63	62	61	61	60	61	62	62	58	54	55	53
	>75mmol/mol (%) (9%)	33	33	33	32	31	31	32	31	32	29	26	27	26
	>86mmol/mol (%) (10%)	14	15	14	14	14	13	14	15	14	13	12	12	11
	N	3598	3846	3902	4003	4126	4155	4247	4317	4274	4462	4600	4511	3788

ESM Fig 1: Estimated proportion and 95% confidence intervals of people with type 1 diabetes in Scotland with good glycaemic control (a), stratified by age (b), sex (c) and SIMD quintile (where 1 is the most deprived quintile) (d). Good glycaemic control was defined as ≤ 58mmol/mol in patients aged ≤ 18 years and 53mmol/mol in patients aged >18 years.

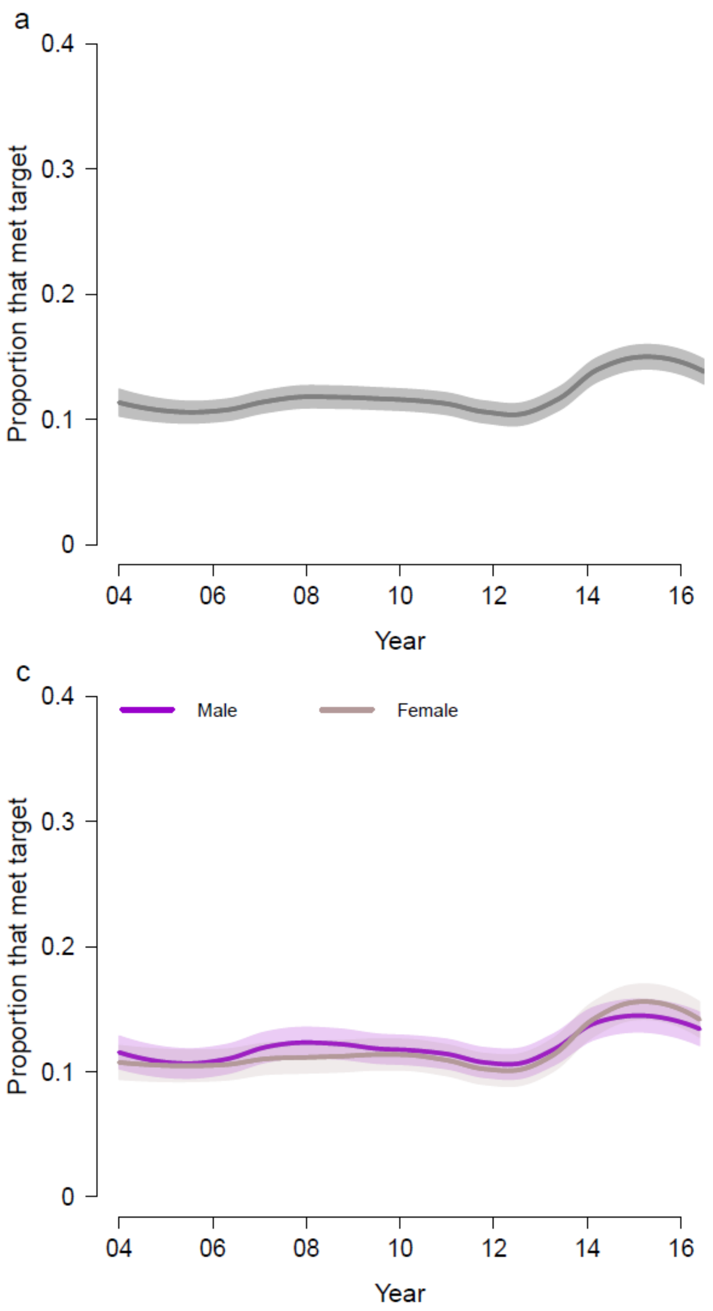

ESM Fig 2: Estimated proportion and 95% confidence intervals of people with type 1 diabetes in Scotland with good glycaemic control (a), stratified by age (b), sex (c) and SIMD quintile (where 1 is the most deprived quintile) (d). Good control was defined using NICE guidelines of 48mmol/mol.

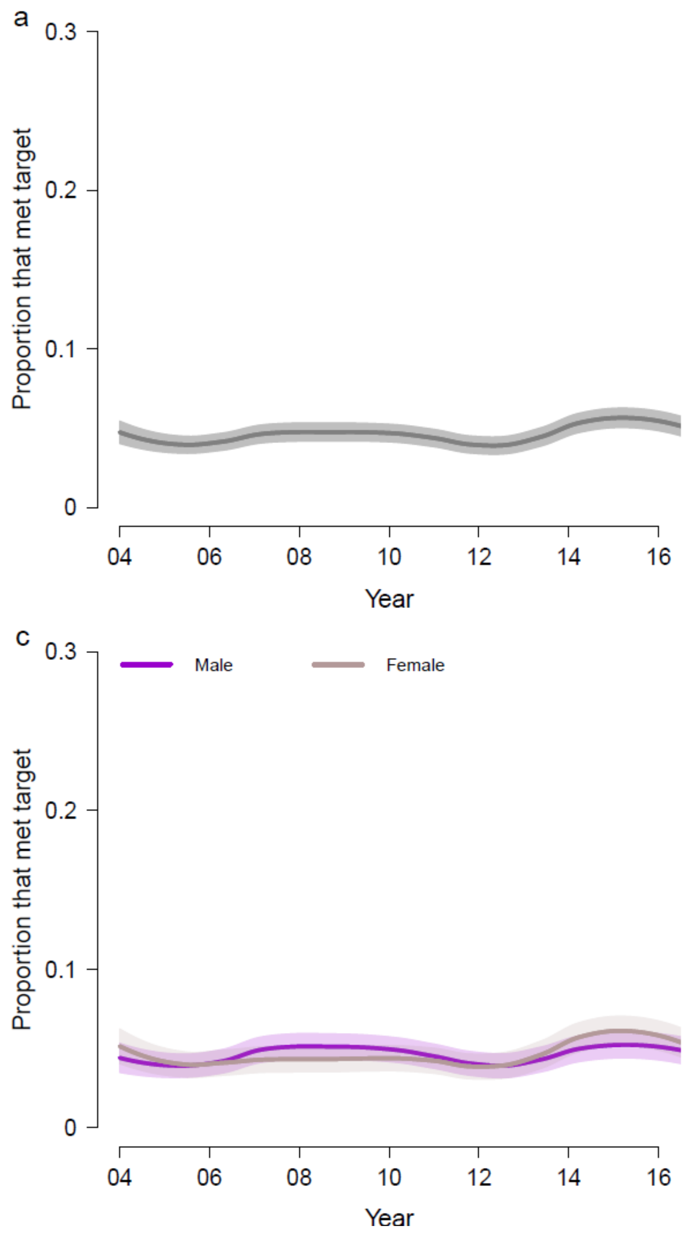

Supplement: Supplementary file 1 — (PDF 473 kb) [file 125_2019_4900_MOESM1_ESM.pdf]
